# Case Report: Case series: association between blood concentration and side effects of sotorasib

**DOI:** 10.3389/fonc.2023.1269991

**Published:** 2023-11-16

**Authors:** Ryota Shigaki, Ryohei Yoshida, Akari Yagita, Kazunori Nagasue, Taeka Naraoka, Kiichi Nitanai, Hiraku Yanada, Toshiyuki Tenma, Ryotaro Kida, Yasuhiro Umekage, Chie Mori, Yoshinori Minami, Hideki Sato, Kuninori Iwayama, Yasuhisa Hashino, Masahide Fukudo, Takaaki Sasaki

**Affiliations:** ^1^ Respiratory Center, Asahikawa Medical University, Hokkaido, Japan; ^2^ Department of Respiratory Medicine, Yoshida Hospital, Hokkaido, Japan; ^3^ Department of Pharmacotherapy, Hokkaido University of Science, Hokkaido, Japan; ^4^ Department of Pharmacy, Sapporo Medical University Hospital, Hokkaido, Japan

**Keywords:** blood level, Kirsten rat sarcoma viral oncogene homologue, patients, side effect, sotorasib

## Abstract

**Introduction:**

Sotorasib is a crucial therapeutic agent for patients with non-small cell lung cancer (NSCLC) harboring the KRAS p.G12C mutation. Despite its efficacy, the relationship between blood sotorasib concentrations and side effects remains largely unexplored.

**Methods:**

This study enrolled five patients with KRAS p.G12C-positive NSCLC treated with sotorasib (LUMAKRAS^®^ Tablets, Amgen, Japan) between July 2022 and February 2023 at Asahikawa Medical University Hospital. Blood sotorasib levels were monitored, and their association with adverse events was examined, with no adjustments made to drug dosages based on these levels.

**Results:**

Variable blood sotorasib levels were observed among the participants. Notably, one patient developed interstitial pneumonitis, although a definitive attribution to sotorasib was uncertain due to prior pembrolizumab treatment. The study revealed no consistent association between blood sotorasib levels and adverse events or therapeutic outcomes, with some patients experiencing severe side effects at higher concentrations, while others did not.

**Conclusion:**

Preliminary findings suggested that monitoring blood sotorasib levels may aid in anticipating adverse events in this small cohort. However, future studies with larger sample sizes and extended follow-up periods are required to validate these initial observations. Such studies could potentially offer insights into personalized dosing strategies, thereby mitigating adverse effects and enhance patient care for individuals with KRAS p.G12C-positive NSCLC.

## 1 Introduction

Activating mutations in the Kirsten rat sarcoma viral oncogene homologue (*KRAS*) are frequently reported in human cancers (1). Among *KRAS* mutations, the p.G12C mutation occurs in 13–15% of patients with non-small cell lung cancer (NSCLC) (2–4). Sotorasib (AMG510) is a small molecule that irreversibly and selectively inhibits *KRAS* p.G12C tumors (5).

Sotorasib has been reported to significantly increase progression-free survival in patients with advanced NSCLC harboring the *KRAS* p.G12C mutation who had been previously treated with other anticancer drugs. While most adverse events associated with sotorasib were tolerable, 56 of 171 (33%) patients experienced grade 3 or worse adverse events that required drug discontinuation or dose reduction (6). As adverse events are evaluated based on the Common Terminology Criteria for Adverse Events (CTCAE), nausea and gastrointestinal symptoms (which are frequently observed with sotorasib) may not be accurately reflected because they are based on subjective findings, unlike objective findings, such as blood toxicity. Therefore, early detection and management of these adverse events are important for the optimal use of sotorasib.

Although previous studies have indicated an association between blood afatinib maleate levels and treatment-related adverse events in NSCLCs exhibiting epidermal growth factor receptor (*EGFR*) mutations (7, 8), the association between sotorasib blood concentration and treatment-related adverse events in patients with NSCLC with the *KRAS* p.G12C mutation has not been previously reported. This study investigated the association between sotorasib blood concentration and related side effects in five patients with NSCLC harboring the *KRAS* p.G12C mutation.

## 2 Case description

### 2.1 Patients

This study included patients who received sotorasib (LUMAKRAS® Tablets, Amgen, Japan) for the treatment of NSCLC at Asahikawa Medical University Hospital between July 2022 and February 2023. All five patients were diagnosed with KRAS p.G12C-positive NSCLC using the Therascreen polymerase chain reaction kit (QIAGEN, Hilden, Germany).

### 2.2 Sotorasib administration

Sotorasib was administered at 960 mg/day. When grade ≥3 side effects were observed, sotorasib administration was discontinued. When the side effect severity decreased to grade 1, the dose was reduced by half, and sotorasib administration was restarted. Administration was discontinued in cases of pneumonitis. The minimum sotorasib dose was 240 mg/day.

### 2.3 Evaluation of sotorasib efficacy and side effects

The chief physician evaluated sotorasib effectiveness based on the Response Evaluation in Solid Tumors (Japan Oncology Group version) criteria. Sotorasib-related side effects were assessed according to CTCAE version 5.0.

### 2.4 Determination of blood sotorasib (AMG 510) levels

Blood samples were collected at least 24 h after the last dose of sotorasib. Blood was collected and centrifuged for the immediate preparation of serum, which was stored at -80°C until analysis. Serum AMG 510 levels were measured using a liquid chromatography-tandem mass spectrometer (API 3200 LC-MS/MS system, Framingham, MA, USA), according to a previous report (9). Chromatographic separation of AMG 510 and internal standard (IS) was achieved on an L-column3 C18 (Chemicals Evaluation and Research Institute, Tokyo, Japan) column (50 × 2.1 mm, 3 μm) maintained at 40°C using a mobile phase containing 0.2% formic acid and acetonitrile (25:75, v/v). The flow rate was 0.65 mL/min, and the injection volume was 10 μL. Mass spectrometry was performed using positive electrospray ionization for quantification of AMG 510 and IS. Detection was via multiple reaction monitoring. Mass transitions (precursor ion-product ion) m/z 561.3–133.9 and 566.3–98.1 were monitored for AMG 510 and IS, respectively. The lower limit of quantification was set at 50 ng/mL, and the final value was corrected with a calibration curve.

### 2.5 Patient characteristics

**Figure 1:**
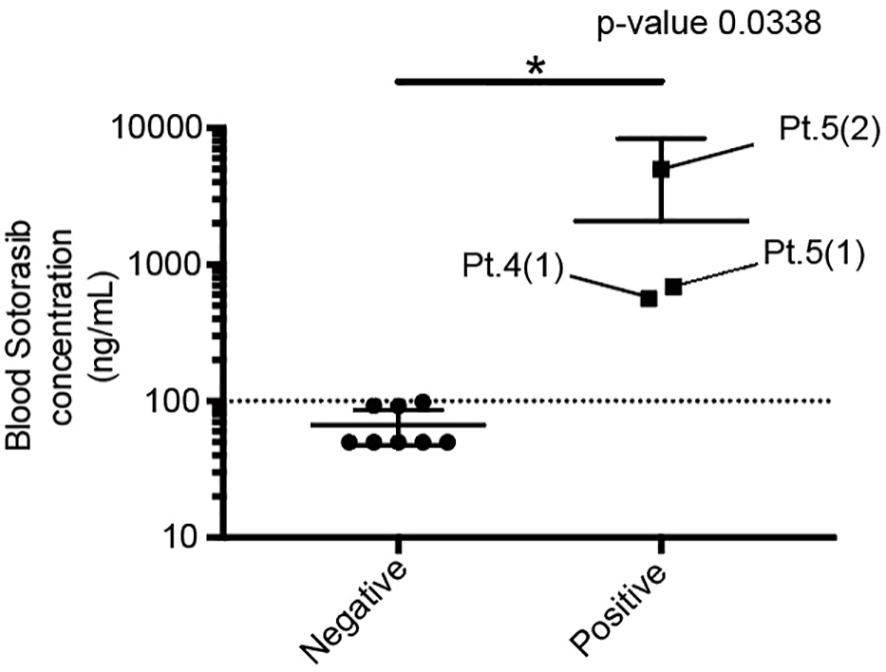
Comparison of the blood sotorasib concentration between the positive and negative groups. Each of the three patients in the positive group was assigned a patient number (Pt.). The numbers in parentheses represent the sequence of blood samples taken from the patients. *P < 0.05.

The patient characteristics are presented in **Table 1**. There were three and two female and male patients, respectively (mean age, 79.6 years). The mean height, weight, body surface area, and body mass index were 153.9 cm, 51.6 kg, 1.47 m^2^, and 21.9 kg/m^2^, respectively. All patients received sotorasib as a second-line therapy. The median follow-up duration was 7.0 months. Sotorasib administration resulted in a partial response in four patients and stable disease in one patient with an overall response rate of 80%. A total of 11 samples were collected from the five patients, and the blood sotorasib levels were analyzed. The values after correction by the calibration curve were defined as negative when <100 ng/mL and positive when ≥100 ng/mL. The data obtained below the lower limit of quantification were treated as 50 ng/mL. The mean and 95% confidence interval (CI) for each group were as follows: mean ± standard error of the mean (SEM) for the positive group was 2085 ± 1460, n=3; mean ± SEM for the negative group was 66.64 ± 8.147, n=8 (95% CI, 192.2–3845) (**Figure 1**). Patients 1, 2, and 3 exhibited blood sotorasib levels <100 ng/mL. Patients 1 and 2 did not experience side effects that necessitated sotorasib discontinuation (**Figure S1**, **2**). Although Patient 3 developed drug-induced interstitial pneumonitis and required the discontinuation of sotorasib, this side effect could not be conclusively attributed to sotorasib due to prior pembrolizumab therapy. Despite sotorasib levels being below the detection sensitivity in blood samples taken after discontinuation, pembrolizumab (up to 3.504 μg/m) was detected in Patient 3’s samples, even though it had been approximately 6 months since the last dose (**Figure S3**). Patient 4 exhibited a high blood level of 565.5 ng/ml of sotorasib in a blood draw 4 weeks after initiation, with no apparent side effects observed during the observation period (**Figure S4**).

**Table 1:** Patient background characteristics.

Case	1	2	3	4	5
Age (years)	84	82	76	78	78
Sex	Female	Female	Male	Male	Female
Height (cm)	144.2	154.2	161.5	162.8	147
Weight (kg)	56.5	40	66.9	44.9	49.8
BSA (m^2^)	1.46	1.329	1.71	1.45	1.41
BMI (kg/m^2^)	27.2	16.8	25.6	16.9	23
Performance status	0	0	0	3	0
Histology	Adeno	Adeno	Adeno	Sarcomatoid carcinoma	Adeno
*KRAS* mutation	G12C	G12C, G13C	G12C	G12C	G12C
PD-L1 (22C3)	<1%	5%	20%	<1%	<1%
Clinical stage	IVA	IVB	IIIC	IVB	IVB
Metastasis	Pulmonary	Pulmonary bone	–	Brain, bone, gastric, adrenal gland, skin	Bone
Treatment line	2	2	2	2	2
First-line treatment	PEM	PEM	CBDCA+PEM+Pemb	CBDCA+PTX+Pemb	CBDCA+PEM
Sotorasib administration period (days)	211	210	277	On-going	134
PFS (months)	6.9	4	9	2	4.6
OS (months)	6.9	4	9	2.5	5.4
eGFR (mL/min/1.73 m^2^)	68	54.6	47.1	95.4	35.2
Alb (IU/L)	3.8	3.7	4.1	3.1	3.5
Best overall response	PR	PR	PR	SD	PR
Side effects	none	none	interstitial pneumonitis (suspicion of pembrolizumab)Grade 3	none	nausea/vomiting Grade 2 fatigue Grade 4

Adeno, adenocarcinoma; Alb, albumin; BMI, body mass index; BSA, body surface area; CBDCA, carboplatin; eGFR, estimated glomerular filtration rate; KRAS, Kirsten rat sarcoma viral oncogene homologue; OS, overall survival; PD-L1, programmed death-ligand 1; PEM, pemetrexed; Pemb, pembrolizumab; PFS, progression-free survival; PR, partial response; PTX, paclitaxel; SD, stable disease.

### 2.6 Treatment course in patient 5

**Figure 2:**
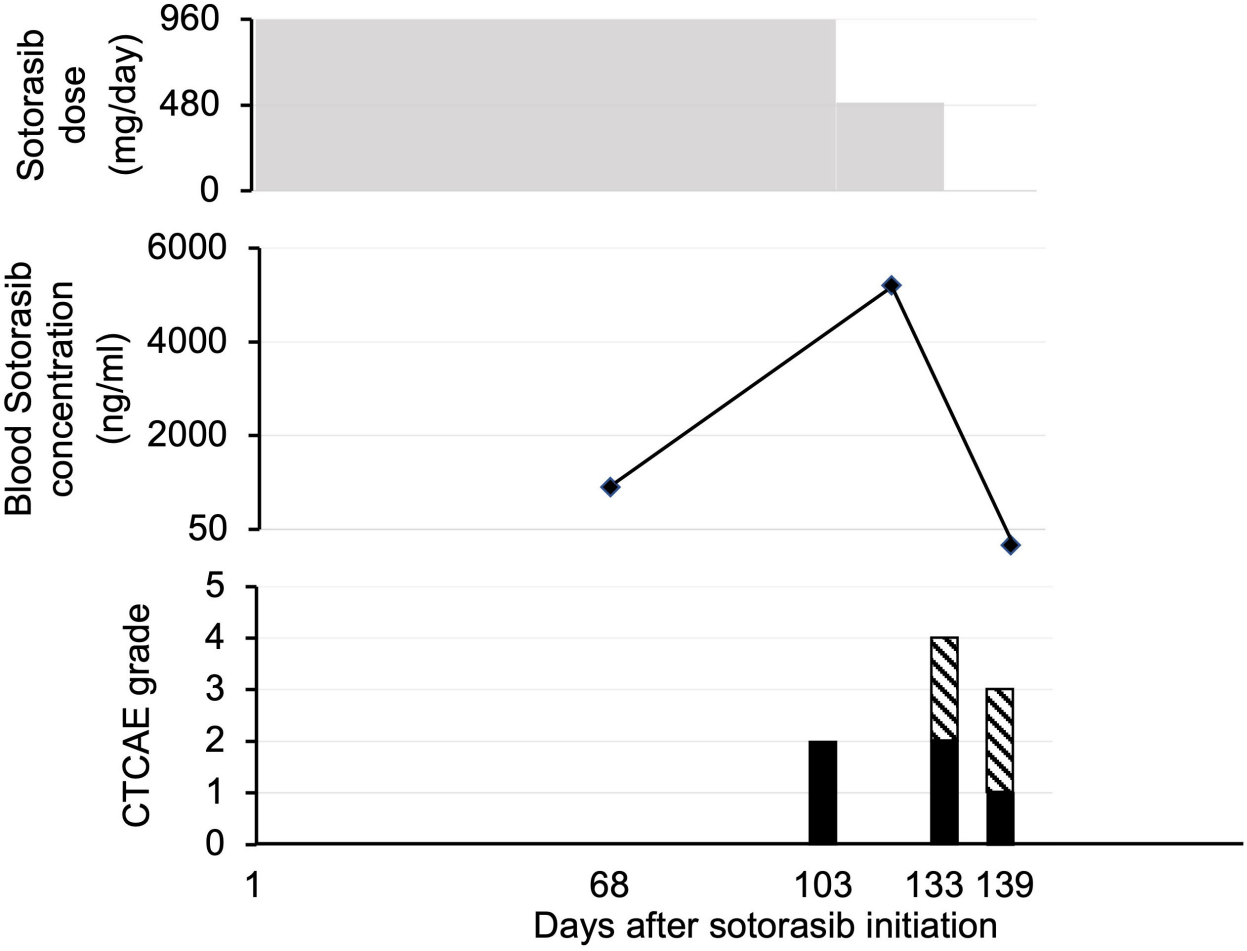
Patient 5. Course of sotorasib administration, blood sotorasib levels, and associated adverse events. In the side effect graph, the black bar represents nausea/vomiting, while the hatched pattern indicates fatigue. CTCAE, Common Terminology Criteria for Adverse Events.

Patient 5 was a 78-year-old woman who presented with pneumonia that did not respond to antibiotics. Computed tomography (CT) revealed consolidation of the right lung; a biopsy was performed, and the diagnosis was invasive mucinous adenocarcinoma (cT4N0M0 c Stage IIIA harboring the *KRAS* p.G12C mutation and programmed death-ligand 1 [22C3] positivity at <1%). The patient was initially treated with carboplatin and pemetrexed. After three treatment cycles, renal dysfunction occurred, and chemotherapy was discontinued. Nine weeks after the last dose of chemotherapy, CT revealed an enlarged right lung lobe cancer, new metastatic mediastinal lymph nodes, and bone metastases. Four months after the initiation of the first treatment, second-line sotorasib therapy (960 mg once daily) was initiated. Eleven days after the initiation of oral medication, a chest radiograph showed tumor regression in the right lung, indicating a partial response (**Figure 2**). The blood sotorasib level on day 68 after administration was 656 ng/mL. On day 103 of sotorasib administration, the patient experienced grade 2 nausea; therefore, the dose was subsequently reduced to 480 mg once daily. Despite dose reduction, blood sotorasib levels were as high as 5055 ng/mL 3 weeks later. She concurrently took multiple oral medications during the period of treatment with sotorasib, but no medications were identified as suspects for elevating her blood levels.

Sotorasib administration was terminated 4 weeks after dose reduction owing to disease progression in the re-enlarged mediastinal lymph nodes. The blood sotorasib level at 10 days after discontinuation was <50 ng/mL. At 4.5 months after sotorasib treatment, the patient was hospitalized for pneumonia and severe dehydration. Despite therapeutic intervention after admission, the patient’s general condition gradually deteriorated, and the patient died 5.3 months after sotorasib treatment. The total duration of sotorasib treatment was 134 days. A pathological autopsy was performed, and the results confirmed death due to advanced lung cancer.

## 3 Discussion

To our knowledge, no prior studies have examined the association between the blood sotorasib levels and side effects in patients with *KRAS* p.G12C-positive NSCLC in a clinical setting in Japan. Notably, drug dosages were not adjusted based on blood sotorasib levels as there is currently no evidence to support this approach.

We observed variable blood sotorasib levels among the five patients. The approved starting dose of sotorasib was 960 mg once daily. The pharmacokinetic profile of this regimen was as follows: the maximum plasma concentration was 7500 ng/mL (coefficient of variation: 98.3%), the median time to maximum plasma concentration was 2.0 (range: 0.3–6.0) h, and the mean (± standard deviation) elimination half-life was 5.5 ± 1.8 h (10). Patients 4 and 5 exhibited blood sotorasib levels >500 ng/mL 24 h after the dose. While Patient 5 experienced grade 2 nausea and fatigue, which led to sotorasib discontinuation, Patient 4 did not report any severe adverse events. In the CodeBreaK100 trial, treatment-related side effects led to dose modification of sotorasib (dose interruption, reduction, or both) in 22.2% of the patients and therapy discontinuation in 7.1% (11). We identified the potential utility of measuring blood sotorasib levels (particularly blood trough levels) after administration, which might serve as a predictor of side effects. Further studies are needed to validate this hypothesis and examine whether dose reduction or discontinuation is warranted in cases with a significant elevation in blood sotorasib levels. In addition, it will be important in future studies to ascertain the appropriate timing for the initial blood draw when monitoring potential side effects.

The study limitations include the single-center design, small sample size, and short follow-up duration. Consequently, further investigations with larger patient cohorts and extended follow-up durations are necessary to validate our findings and expand the potential clinical applications of blood sotorasib level monitoring.

In conclusion, the assessment of blood sotorasib concentrations is beneficial for monitoring adverse events as they may serve as a predictive marker for the emergence of side effects. If this hypothesis is valid, it would be advisable to consider dose reduction or discontinuation in cases where a significant elevation in blood sotorasib levels is observed.

## Data availability statement

The raw data supporting the conclusions of this article will be made available by the authors, without undue reservation.

## Ethics statement

The study protocol was approved by the institutional review board of Asahikawa Medical University on July 11, 2022 (approval number: 22031) and conducted in accordance with the principles of the Declaration of Helsinki. All patients provided informed consent for blood collection and the use of their clinical data for analysis and publication. Written informed consent was obtained from the patients/participants for the publication of this case report.

## Author contributions

RS: Data curation, Visualization, Writing – original draft. RY: Conceptualization, Methodology, Supervision, Visualization, Writing – review & editing. AY: Investigation, Writing – review & editing. KaN: Investigation, Writing – review & editing. TN: Investigation, Writing – review & editing. HY: Investigation, Writing – review & editing. KiN: Investigation, Resources, Writing – review & editing. TT: Investigation, Resources, Writing – review & editing. RK: Investigation, Writing – review & editing. YU: Investigation, Writing – review & editing. CM: Investigation, Resources, Writing – review & editing. YM: Investigation, Supervision, Writing – review & editing. HS: Formal Analysis, Methodology, Writing – review & editing. KI: Formal Analysis, Methodology, Writing – review & editing. YH: Formal Analysis, Methodology, Writing – review & editing. MF: Formal Analysis, Methodology, Supervision, Writing – review & editing. TS: Conceptualization, Project administration, Writing – review & editing.

## Supplementary material

Supplementary Figure 1 Patient 1. Course of sotorasib administration, blood sotorasib levels, and associated adverse events. Patient 1 was transferred to another hospital 14 days after the initiation of sotorasib treatment; hence, we were unable to track their subsequent clinical progress.

Supplementary Figure 2 Patient 2. Course of sotorasib administration, blood sotorasib levels, and associated adverse events. It has been over a year since the initiation of sotorasib; however, Patient 2 did not experience any adverse events.

Supplementary Figure 3 Patient 3. The course of sotorasib dose, blood sotorasib levels, and associated adverse event. In the side effect graph, shaded bars indicate interstitial pneumonitis.

Supplementary Figure 4 Patient 4. Course of sotorasib administration, blood sotorasib levels, and associated adverse events. Patient 4 did not experience any adverse events.
