# Supplementary figures and images for: Case Report: Case series: association between blood concentration and side effects of sotorasib
Source: Front Oncol. 2023 Nov 16;13:1269991. doi: 10.3389/fonc.2023.1269991 (PMC10690615; doi:10.3389/fonc.2023.1269991)

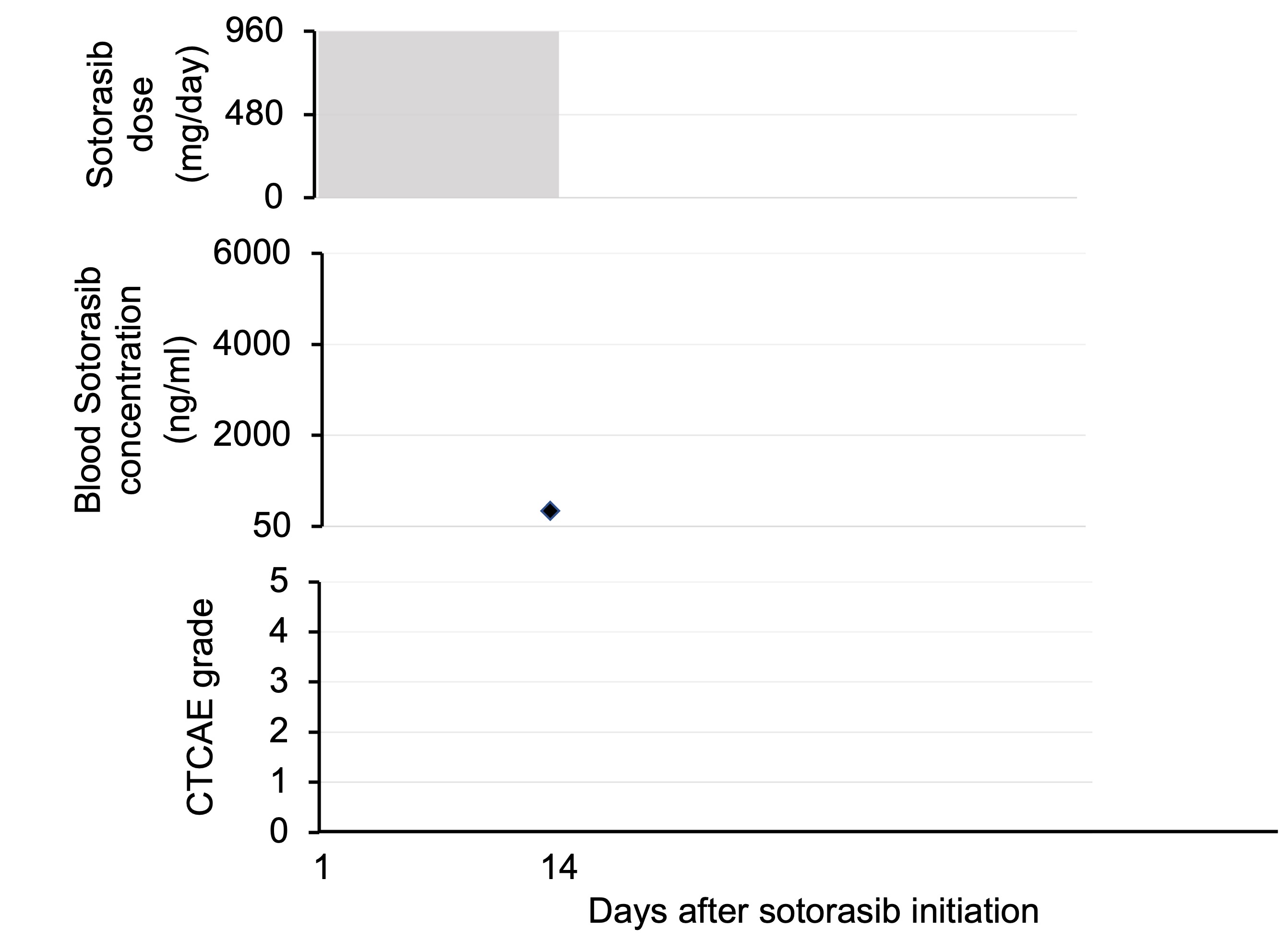

Supplement: Supplementary Figure 1 — Patient 1. Course of sotorasib administration, blood sotorasib levels, and associated adverse events. Patient 1 was transferred to another hospital 14 days after the initiation of sotorasib treatment; hence, we were unable to track their subsequent clinical progress. [file Image_1.jpeg]

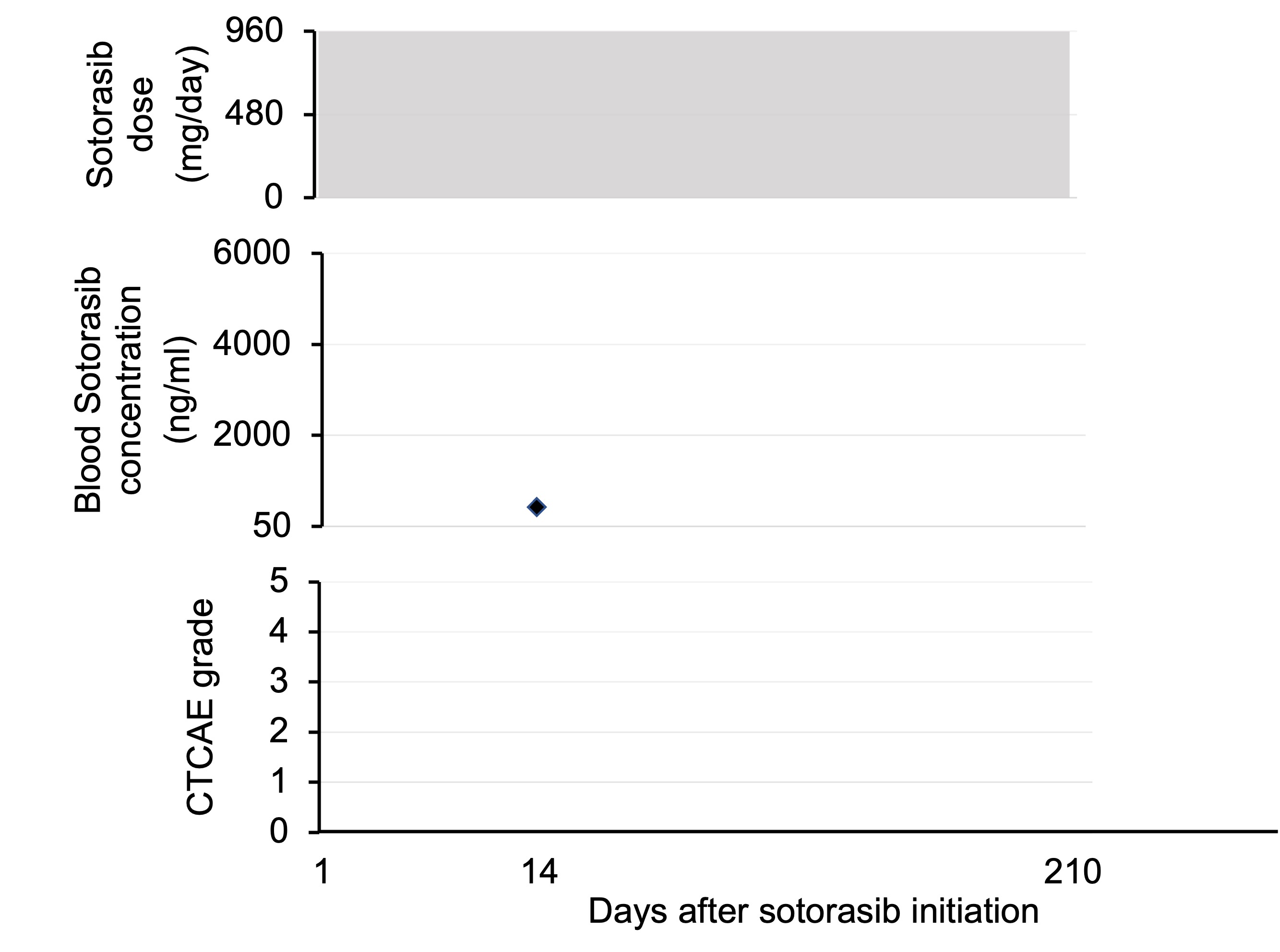

Supplement: Supplementary Figure 2 — Patient 2. Course of sotorasib administration, blood sotorasib levels, and associated adverse events. It has been over a year since the initiation of sotorasib; however, Patient 2 did not experience any adverse events. [file Image_2.jpeg]

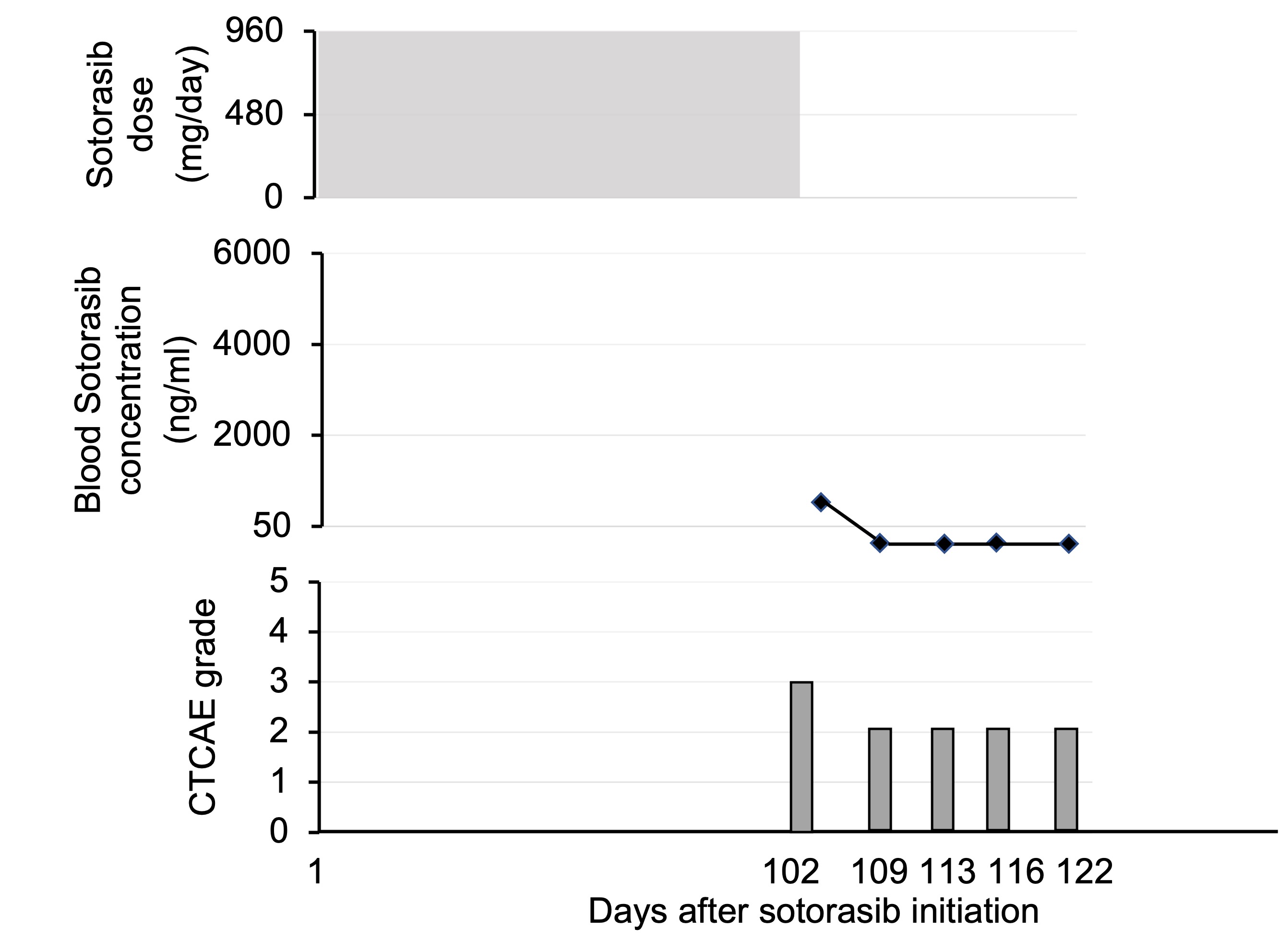

Supplement: Supplementary Figure 3 — Patient 3. The course of sotorasib dose, blood sotorasib levels, and associated adverse event. In the side effect graph, shaded bars indicate interstitial pneumonitis. [file Image_3.jpeg]

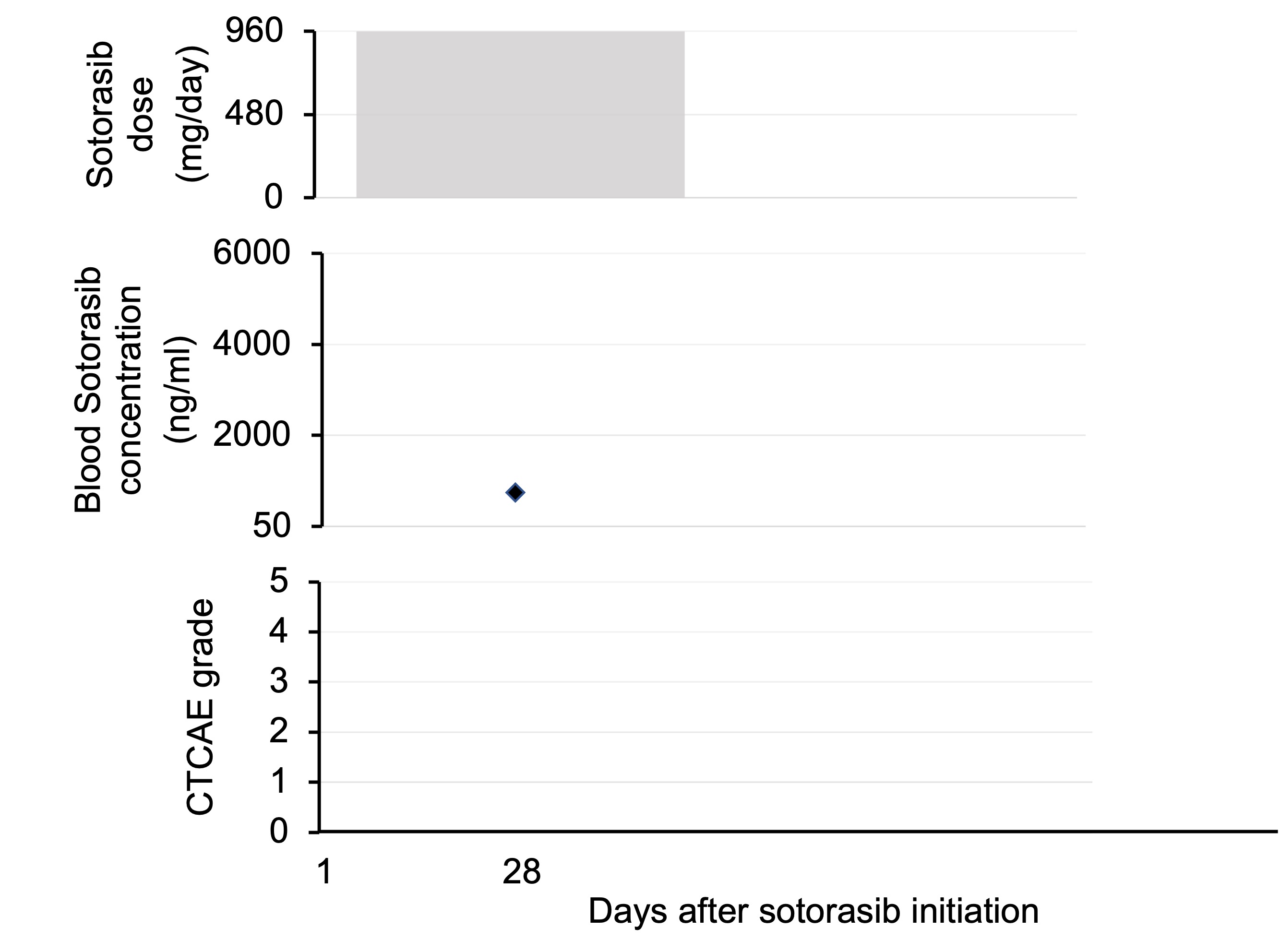

Supplement: Supplementary Figure 4 — Patient 4. Course of sotorasib administration, blood sotorasib levels, and associated adverse events. Patient 4 did not experience any adverse events. [file Image_4.jpeg]
